# Functional Genes and Bacterial Communities During Organohalide Respiration of Chloroethenes in Microcosms of Multi-Contaminated Groundwater

**DOI:** 10.3389/fmicb.2019.00089

**Published:** 2019-02-12

**Authors:** Louis Hermon, Jennifer Hellal, Jérémie Denonfoux, Stéphane Vuilleumier, Gwenaël Imfeld, Charlotte Urien, Stéphanie Ferreira, Catherine Joulian

**Affiliations:** ^1^Geomicrobiology and Environmental Monitoring Unit, Bureau de Recherches Géologiques et Minières (BRGM), Orléans, France; ^2^CNRS, GMGM UMR 7156, Genomics and Microbiology, Université de Strasbourg, Strasbourg, France; ^3^Service Recherche, Développement et Innovation-Communautés Microbiennes, GenoScreen, SAS, Lille, France; ^4^CNRS/EOST, LHyGeS UMR 7517, Laboratory of Hydrology and Geochemistry of Strasbourg, Université de Strasbourg, Strasbourg, France

**Keywords:** perchloroethylene (PCE), chloroethenes (CEs), contaminated groundwater, dehalogenase genes, organohalide respiration

## Abstract

Microcosm experiments with CE-contaminated groundwater from a former industrial site were set-up to evaluate the relationships between biological CE dissipation, dehalogenase genes abundance and bacterial genera diversity. Impact of high concentrations of PCE on organohalide respiration was also evaluated. Complete or partial dechlorination of PCE, TCE, *cis*-DCE and VC was observed independently of the addition of a reducing agent (Na_2_S) or an electron donor (acetate). The addition of either 10 or 100 μM PCE had no effect on organohalide respiration. qPCR analysis of reductive dehalogenases genes (*pceA, tceA, vcrA*, and *bvcA*) indicated that the version of *pceA* gene found in the genus *Dehalococcoides* [hereafter named *pceA*(Dhc)] and *vcrA* gene increased in abundance by one order of magnitude during the first 10 days of incubation. The version of the *pceA* gene found, among others, in the genus *Dehalobacter, Sulfurospirillum, Desulfuromonas*, and *Geobacter* [hereafter named *pceA*(Dhb)] and *bvcA* gene showed very low abundance. The *tceA* gene was not detected throughout the experiment. The proportion of *pceA*(Dhc) or *vcrA* genes relative to the universal 16S ribosomal RNA (16S rRNA) gene increased by up to 6-fold upon completion of *cis-*DCE dissipation. Sequencing of 16S rRNA amplicons indicated that the abundance of Operational Taxonomic Units (OTUs) affiliated to dehalogenating genera *Dehalococcoides, Sulfurospirillum*, and *Geobacter* represented more than 20% sequence abundance in the microcosms. Among organohalide respiration associated genera, only abundance of *Dehalococcoides* spp. increased up to fourfold upon complete dissipation of PCE and *cis*-DCE, suggesting a major implication of *Dehalococcoides* in CEs organohalide respiration. The relative abundance of *pceA* and *vcrA* genes correlated with the occurrence of *Dehalococcoides* and with dissipation extent of PCE, *cis*-DCE and CV. A new type of dehalogenating *Dehalococcoides* sp. phylotype affiliated to the Pinellas group, and suggested to contain both *pceA*(Dhc) and *vcrA* genes, may be involved in organohalide respiration of CEs in groundwater of the study site. Overall, the results demonstrate *in situ* dechlorination potential of CE in the plume, and suggest that taxonomic and functional biomarkers in laboratory microcosms of contaminated groundwater following pollutant exposure can help predict bioremediation potential at contaminated industrial sites.

## Introduction

Extensive industrial use of halogenated volatile organic compounds (VOC) such as tetrachloroethylene (PCE) and trichloroethylene (TCE) has resulted in widespread environmental contamination of soil and groundwater worldwide (Huang et al., 2014). Groundwater at industrial sites producing or using halogenated VOC is often contaminated by chlorinated ethenes (CEs), generally in mixture with other contaminants (Aktaş et al., 2012). Once released into the environment, CEs migrate through the unsaturated zone of the subsurface (vadose zone), and can accumulate in aquifers as a dense non-aqueous phase liquid (DNAPL) due to their solubility, density, and hydrophobicity. Diffusion and transport of CEs in groundwater may result in a contamination plume often characterized by pollutant and redox gradients determining *in situ* biotransformation (Haack et al., 2004).

Organohalide respiration of CEs occurs under both oxic and anoxic conditions (Vogel and McCarty, 1985; Freedman and Gossett, 1991; Maymó-Gatell et al., 1999; Beeman and Bleckmann, 2002; Atashgahi et al., 2017). Under anoxic conditions, CEs may serve as electron acceptors for microbial metabolism (Holliger et al., 1998). Upon reductive dechlorination, PCE can be enzymatically converted, sequentially to TCE, *cis*-DCE, VC, and finally to non-toxic ethene. As the number of chlorine substituents decreases, dechlorination rate usually slows down with accumulation of less chlorinated DCE and VC (Abe et al., 2009; Chang et al., 2017).

**FIGURE 1:**
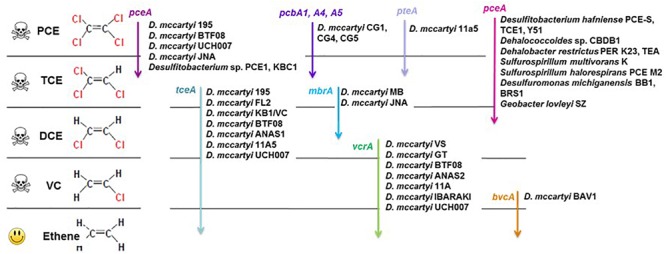
Organohalide respiration pathways of chloroethenes, associated bacteria and reductive dehalogenase genes (based on Futagami et al., 2008; Hug, 2016; Zhao et al., 2017; Saiyari et al., 2018).

Bioremediation and monitored natural attenuation are promising approaches for monitoring and removing chlorinated solvents from contaminated aquifers, due to their efficiency, sustainability and relatively low costs (Da Silva and Alvarez, 2008; Kang, 2014; Patil et al., 2014). Biostimulation studies for field remediation of chlorinated ethenes have been recently reported (Santharam et al., 2011; Florey et al., 2017; Sheu et al., 2018). Many bacterial strains capable of reductive dehalogenation of CEs have been characterized (Atashgahi et al., 2016). They typically belong to the genera *Dehalococcoides, Dehalobacter, Desulfitobacterium, Sulfurospirillum*, and *Geobacter.* These genera have thus been suggested as potential bioindicators of dechlorination in aquifers (Löﬄer et al., 2000; Hendrickson et al., 2002; Duhamel and Edwards, 2006; Imfeld et al., 2008; Clark et al., 2018). While many strains of organohalide respiration associated genera are involved in one or several organohalide respiration steps of halogenated VOC, complete reductive dehalogenation of PCE to ethene has only been observed for some strains of the *Dehalococcoides* genus (Maymó-Gatell et al., 2001; He et al., 2003; Muller et al., 2004; Sung et al., 2006b). Dechlorinating capacities of *Dehalococcoides* strains may, however, differ widely (Magnuson et al., 1998; Lee et al., 2008). At the functional level, various genes encoding different types of reductive dehalogenases are involved in specific steps of sequential CE dechlorination (Figure 1) (Futagami et al., 2008; Hug, 2016; Saiyari et al., 2018). The dehalogenation of PCE to TCE and DCE (mainly *cis*-1,2-DCE) by non-obligatory dehalogenating bacteria is encoded by *pceA* genes. In *Dehalococcoides*, PCE is dechlorinated to TCE by strains also carrying a *pceA* gene (Magnuson et al., 1998). More recent findings highlighted the diversity of genes encoding PCE reductive dehalogenase in *Dehalococcoides*; *pteA* gene (PCE to TCE) in *Dehalococcoides mccartyi* strain 11a5 (Zhao et al., 2017), *pcbA* genes (PCE to TCE and DCE) in three distinct PCB-dechlorinating *Dehalococcoides* strains (Wang et al., 2014), and *mbrA* gene in strain MB that produce mainly *trans*-1,2-DCE (Cheng and He, 2009; Chow et al., 2010). The next steps, i.e., TCE to DCE, VC and ethene, only occur in *Dehalococcoides* strains and are encoded by three genes, *tceA* (TCE to DCE), *vcrA* or *bvcA* (DCE to CV and ethene) (Zinder, 2016).

Knowledge of microbial and gene diversities associated with organohalide respiration of CEs has contributed to develop biomolecular approaches to evaluate biological dehalogenation and guide remediation strategies at contaminated sites (Maphosa et al., 2010; Dugat-Bony et al., 2012). Such approaches mainly rely on sensitive detection and quantification of specific dehalogenating taxa, notably *Dehalococcoides* (Hendrickson et al., 2002; Lu et al., 2006; Rouzeau-Szynalski et al., 2011; Kranzioch et al., 2013) or of functional genes used as biomarkers of dehlorespiration (Behrens et al., 2008; Da Silva and Alvarez, 2008; Rahm and Richardson, 2008; Carreon-Diazconti et al., 2009; Kranzioch et al., 2013). However, interpretation of currently available biomarkers and identification of novel ones requires a better understanding of the relationship between bacterial taxa associated with organohalide respiration of CEs and genes involved in dechlorination steps (Maphosa et al., 2012).

Here we examined the potential of endogenous groundwater bacterial communities of a former industrial site contaminated with CEs to degrade PCE and other CEs in relation to bacterial diversity and functional genes associated with reductive dechlorination of CEs. The aim was to evaluate the relationships between biological CE dissipation, dehalogenase gene abundance and bacterial genera diversity that could be transposed as biomarkers to the field. The effect of high additions of PCE on organohalide respiration rate and associated bacterial diversity was also addressed. The experimental setup consisted of laboratory microcosms containing groundwater from a multi-contaminated former industrial site. The experimental set-up was designed to evaluate the effect of PCE concentration, and of addition of a carbon source (acetate) and a reducing agent (Na_2_S) on changes of specific genera associated with organohalide respiration, and reductive dehalogenases genes *pceA, tceA, vcrA*, and *bvcA*.

## Materials and Methods

### Groundwater Sampling

Groundwater samples were taken from the former industrial site of Themeroil (Varennes-le-Grand, France, GPS coordinates, 46.701141 N, 4.843919 E) characterized by historical intensive oil and solvent processing activities. Halogenated solvents and BTEX were released into the site aquifer due to inappropriate storage methods (BRGM, 1998), resulting in high concentrations of chlorinated VOC (mainly CEs) and BTEX, accumulated within DNAPL in the groundwater of the site. The pollutant plume extends over an area of about 5 ⋅ 10^-2^ km^2^ (BRGM, 2011). Low concentrations of PCE (20 μg ⋅ L^-1^) and TCE (11 μg ⋅ L^-1^) in the contaminant plume compared to the contamination source and the prevalence of *cis-* over *trans*-DCE (Table 1) suggest the production of *cis*-DCE from PCE and TCE reductive dehalogenation and its accumulation (Nijenhuis et al., 2007).

**Table 1:** Hydrochemical characteristics of groundwater from well Pz6(10) (June 2015).

Halogenated VOCs	Value (μg ⋅ L^-1^)	Hydrochemistry	Value (unit)
Perchloroethylene	20	pH	6.9
Trichloroethylene	11	Electric conductivity	1833 (μS ⋅ cm^-1^)
*Cis*-1,2-dichloroethylene	34800	Temperature	11.7 (°C)
*Trans*-1,2-dichloroethylene	62	Redox potential	-242 (mV)
Vinyl chloride	7800	Dissolved oxygen	0.05 (mg ⋅ L^-1^)
1,1-dichloroethylene	2284	FeII	3.3 (mg ⋅ L^-1^)
1,2-dichloroethane	53	FeIII	7.4 (mg ⋅ L^-1^)
1,1-dichloroethane	700	SO_4_^2-^	128 (mg ⋅ L^-1^)
Dichloromethane	<10	NO_3_^-^	0.2 (mg ⋅ L^-1^)

Piezometer Pz6(10) located in the contaminant plume (Supplementary Figure S1) was selected based upon favorable redox conditions regarding CEs reductive dehalogenation (dissolved oxygen concentration below 0.05 ppm and redox potential about -242 mV) (Table 1). Major CE contaminants were *cis*-DCE (34.8 mg ⋅ L^-1^) and VC (7.8 mg ⋅ L^-1^). Pz6(10) was characterized by high sulfate (128 mg ⋅ L^-1^) and Fe(III) (7.4 mg ⋅ L^-1^) and low nitrate (<1 mg ⋅ L^-1^) aqueous concentrations. Thirty liters of Pz6(10) groundwater were sampled in June 2015 with a Twister pump (Proactive, Bradenton, United States) at a depth of 5 m, after purging the piezometer and ensuring that *E*_h_, pH, and conductivity were constant. Groundwater was stored for 30 days at 4°C until microcosm set-up.

### Microcosm Set-Up

Pz6(10) groundwater was pre-incubated to favor bacterial reductive dechlorination of PCE and increase reaction kinetics. Pz6(10) groundwater (800 mL) was pre-incubated in a 1 L bottle (Schott DURAN^®^, Germany) with 8 mL of a 30 g ⋅ L^-1^ NH_4_HCO_3_ solution (0.30 g ⋅ L^-1^ final concentration), 8 mL of a 25 g ⋅ L^-1^ K_2_HPO_4_ solution (0.25 g ⋅ L^-1^), 8 mL of a 1 g ⋅ L^-1^ MgSO_4_ ⋅ 7H_2_O solution (0.1 g ⋅ L^-1^); 800 μL of an 80 g ⋅ L^-1^ CaCl_2_ solution (0.08 g ⋅ L^-1^), 500 μL of a 100 μg.L^-1^ vitamin B12 solution (62 μg ⋅ L^-1^), 2.4 mL of a 1 M sodium acetate solution (3 mM; 246 mg ⋅ L^-1^), 640 μL of a 40 g.L^-1^ sodium sulfide solution (32 mg ⋅ L^-1^), 1 mL of a 20 g.L^-1^ yeast extract solution (25 mg ⋅ L^-1^) and 2.6 μL of pure PCE (99.9%, Sigma-Aldrich) solution (20 μM final concentration). The bottle was hermetically closed with a GL45 teflon-coated bottle cap (Omnifit^®^), conditioned with a H_2_/N_2_ atmosphere (5%/95%) and incubated at 20°C in the dark without shaking. After complete dissipation of PCE to TCE (30 days), this pre-culture served as inoculum to the microcosm experiment.

Microcosms consisted of 800 mL of Pz6(10) groundwater, inoculated with the pre-culture at a ratio of 1:10 in 1 L glass bottles (Schott^®^) capped with GL45 teflon-coated bottle caps (Omnifit^®^). Sample handling was performed in a glovebox under a nitrogen atmosphere. Two sets of microcosms were prepared in triplicates with Pz6(10) groundwater and amended with pure PCE (99.9%, Sigma-Aldrich) to obtain final PCE concentrations of, respectively, 10 and 100 μM using a glass syringe (Hamilton Bonaduz AG, Bonaduz, Switzerland). For each condition, microcosms were amended or not with sodium acetate (3 mM final concentration) and sodium sulfide solution (Na_2_S, 0.4 mM final concentration). Killed controls consisted of site water sterilized by autoclaving (twice at 121°C for 30 min with a 24 h break between cycles), with no or 10 μM PCE added after autoclaving. The microcosms were placed under H_2_/N_2_ (5%/95%, 0.5 bar) atmosphere and incubated in the dark at 20°C under agitation (80 rpm). Water samples were taken from microcosms for hydrochemical and DNA analyses immediately after set up (day 0), and after 3, 5, 7, 10, 13, 19, 35, and 55 days of incubation.

### Hydrochemical Analyses

pH and redox potential (*E*_h_) were measured from aqueous phase microcosm aliquots using a portable probe (Multi 340i, WTW Instrument) at all sampling times in a N_2_-purged glove box. Acetate, sulfate, nitrate and chloride were measured at days 5, 10, 19, and 35 in filtered samples (0.22 μm syringe filters, Millipore, United States) by ionic chromatography (Dionex DX-100 equipped with AS19HC column). Quantification limits were 0.1 mg ⋅ L^-1^ for acetate and 0.5 mg ⋅ L^-1^ for sulfate, nitrate and chloride. Sulfide was measured after 10 and 35 days of incubation with a Merck Spectroquant kit 114779 (Merck, Germany).

Concentrations of CEs (PCE, TCE, *cis*-DCE and VC) were determined in 5 mL samples collected at all times. CEs were measured using a gas chromatograph (CP-3008-GC Varian, Walnut Creek, CA, United States) equipped with a headspace sampler and flame ionization detector. Chromatographic separation was performed in a capillary column (Agilent DB-624, 30 m, 0.32 mm inside diameter, 1.80 μm film thickness). Injector and detector temperatures were held at 250 and 300°C, respectively, and the following temperature program was used: hold at 35°C for 5 min, heating to 245°C (10°C/min) and hold for 10 min. Concentrations were determined using external standards (*R*^2^ = 0.99). The limit of quantification was 20 μg ⋅ L^-1^ for all CEs.

Dissipation rates (μM.d^-1^) for individual CE_x_ were estimated as an average of individual time step as follows:

(1) $$\begin{array}{c} {\text{DisCE}}_{\text{x}}[T_{\text{n}};T_{\text{n}}{{}_{\text{-}}}_{\text{1}}]\text{=}([{\text{CE}}_{\text{x}}]T_{\text{n}}-[{\text{CE}}_{\text{x}}]{\text{T}}_{\text{n}}{{}_{\text{-}}}_{\text{1}})-{\text{DisCE}}_{\text{x}}{{}_{\text{+}}}_{\text{1}}[T_{\text{n}};T_{\text{n}}{{}_{\text{-}}}_{\text{1}}] \end{array}$$

Where DisCE is the dissipation rate in μM.d^-1^ and *x* is the number of chlorine atom substituents for each CE_x_ and n the number of incubation days.

### DNA Extraction

Fifteen mL of microcosm groundwater were sampled at each sampling time and filtered through 0.22 μm sterile membrane filters (∅ 2.5 cm, Millipore, United States). Membranes were stored at -20°C until DNA extraction. DNA was extracted using the FastDNA^®^ Spin Kit for Soil (MP Biomedicals, United States) and the Fastprep^®^ instrument according to the manufacturer’s instructions, with minor modifications (30 s lysis at a speed setting of 5.0, subsequent centrifugation of cell debris for 25 min). Extracted total DNA was quantified using the Quantifluor dsDNA sample kit and the Quantus fluorimeter according to the manufacturer’s instructions (Quantus, Promega, United States).

### qPCR Analysis

Quantification of the bacterial 16S rRNA gene and of reductive dehalogenase genes (*pceA, tceA, vcrA*, and *bvcA*) was performed by qPCR using a CFX Connect^TM^ Real-Time PCR Detection System (Bio-Rad, France) with primers and programs listed in Table 2. qPCR reactions were performed in a total volume of 20 μL, with a master mix containing 7.6 μL of RNase- and DNase-free water (MP Biomedicals, CA, United States), 10 μL of SYBR Green IQ Supermix (Bio-Rad), 500 nM of each primer, and 2 μL of template DNA (concentrations of total DNA ranging from 0.1 ng ⋅μL^-1^ to 5 ng ⋅μL^-1^). Sterile, nuclease-, RNA- and DNA-free water was added instead of DNA in no template controls (NTC). All samples, controls and standards were analyzed in duplicate. A calibration curve was obtained from serial dilutions of a known quantity of linearized plasmids containing known copy numbers of 16S *rrnA, pceA*(Dhc), *pceA*(Dhb), *tceA, vcrA*, and *bvcA* gene fragments, respectively. Gene concentrations were reported as gene copies per mL of groundwater preparation. Limits of quantification (LOQ) were 3.2 ⋅ 10^3^ gene copies ⋅ mL^-1^ culture for the 16S rRNA gene, 6.7 ⋅ 10^2^ gene copies ⋅ mL^-1^ for *pceA, tceA*, and *vcrA* genes, and 6.7 ⋅ 10^1^ gene copies ⋅ mL^-1^ for *bvcA*, respectively (Supplementary Table S1). Generation of a specific PCR product was confirmed by melting curve analyses and agarose gel electrophoresis. The effect of PCR inhibitors in DNA was estimated using successive dilutions of the DNA extract mixed with known amounts of DNA standard (pGEM-T easy vector, Promega) for qPCR with vector-specific primers as previously described (Miyata et al., 2010). No PCR inhibition was detected.

**Table 2:** qPCR primers and temperature programs used in this study.

Target gene	Primers	Target	Fragment	Target bacteria	qPCR program	Reference
		sequence 5′–3′	size (bp)			
16S rRNA	341F	CCTACGGGAGGCAGCAG	174	All bacteria	3 min 95°C, 35 cycles: 30 s	López-Gutiérrez et al., 2004
	515R	ATTACCGCGGCTGCTGGCA			95°C/30 s 60°C/30 s 72°C/Melt^a^	
*pceA* (Dhb)	SpDr1f	CGTTGGACCTATTCCACCTG	199	*Dehalobacter restrictus*	3 min 95°C, 40 cycles: 10 s	Regeard et al., 2004
	SpDr1r	CAAGAACGAAGGCAATCACA		PER-K23;	95°C/ 45 s 53°C/30 s 80°C/Melt	
				*Desulfitobacterium hafniense* PCE-S	
*pceA* (Dhc)	pceA877F	ACCGAAACCAGTTACGAACG	100	*Dehalococcoides mccartyi*	3 min 95°C, 40 cycles: 10 s	Behrens et al., 2008
	pceA976R	GACTATTGTTGCCGGCACTT			95°C/45 s 61°C/30 s 80°C/Melt	
*tceA*	tceA511F	GCCACGAATGGCTCACATA	306	*Dehalococcoides mccartyi*;	3 min 95°C, 40 cycles: 10 s	Behrens et al., 2008
	tceA817R	TAATCGTATACCAAGGCCCG		*Dehalococcoides* sp. FL2	95°C/45 s 61°C/30 s 80°C/Melt	
*vcrA*	vcrA880F	CCCTCCAGATGCTCCCTTTA	139	*Dehalococcoides* sp. VS	3 min 95°C, 40 cycles: 10 s	Behrens et al., 2008
	vcrA1018R	ATCCCCTCTCCCGTGTAACC			95°C/45 s 61°C/30 s 80°C/Melt	
*bvcA*	bvcA227F	TGGGGACCTGTACCTGAAAA	247	*Dehalococcoides* sp.	3 min 95°C, 40 cycles: 10 s	Behrens et al., 2008
	bvcA523R	CAAGACGCATTGTGGACATC		BAV-1	95°C/45 s 61°C/30 s 80°C/Melt	

*^*a*^Melt curve acquisition: rising from 65 to 95°C at 0.5°C ⋅ s^-*1*^.*

### CE-SSCP Bacterial Diversity Profiles

For CE-SSCP bacterial community fingerprinting, the V3 region of the 16S rRNA gene was amplified by PCR from DNA extracts with forward primer w49 (5′-ACGGTCCAGACTCCTACGGG-3′) and 5′ FAM-labelled reverse primer w34 (5′-TTACCGCGGCTGCTGGCAC-3′) (Delbès et al., 2001), by 30 s hybridisation at 61°C, and 30 s elongation at 72°C for 28 cycles as described previously. One μL of diluted PCR product (5- to 100-fold in nuclease-free water) was then added to a mixture of 18.6 μL of deionized formamide and 0.4 μL of Genescan-600 LIZ internal DNA standard (Life Technologies, United States). To obtain single-strand DNA, samples were heat-denatured for 10 min at 95°C, and immediately cooled on ice. CE-SSCP analyses were performed on an ABI Prism 310 genetic analyser using a 47-cm long capillary, a non-denaturing 5.6% CAP polymer (Life technologies, United States) and the following electrophoresis conditions: run temperature 32°C, sample injection for 5 s at 15 kV, and data collection for 35 min at 12 kV. Alignment of the profiles using an internal DNA standard and assignment of peak positions were performed with Bionumerics software (Applied Maths, Belgium).

### Illumina MiSeq Sequencing of 16S rRNA Genes

Four samples collected from microcosms on days 0, 5, 10, and 34 were selected for 16S rRNA gene amplicon high-throughput sequencing based on chemical variations and community composition changes as preliminary detected by CE-SSCP analysis according to the distribution and the area of the peaks over time (*p* < 0.05). For each sample, DNA extracted from biological replicates (*n* = 3) was pooled for amplification of V4-V5 hypervariable region (Claesson et al., 2010) using an optimized and standardized amplicon-library preparation protocol (Metabiote^®^, GenoScreen, Lille, France). A positive [artificial bacteria community comprising 17 different bacteria (ABCv2)] and a negative (sterile water) control were also performed. Briefly, PCR reactions were performed using 5 ng of genomic DNA and 192 fusion barcoded primers (at 0.2 μM final concentrations), with an annealing temperature of 50°C for 30 cycles. PCR products were purified using Agencourt AMPure XP magnetic beads (Beckman Coulter, Brea, CA, United States), quantified according to GenoScreen’s protocol, and mixed in an equimolar amount. Sequencing was performed using 250-bp paired-end sequencing chemistry on the Illumina MiSeq platform (Illumina, San Diego, CA, United States) at GenoScreen (Lille, France). The sequence information was deposited to NCBI Sequence Read Archive (SRA) under accession numbers SRP160122.

### Bioinformatic Analysis of 16S rRNA Gene Sequence Data

Raw paired-end reads were demultiplexed per sample and subjected to the following process:

(1) search and removal of both forward and reverse primers using CutAdapt, with no mismatches allowed in the primers sequences;
(2) quality-filtering using the PRINSEQ-lite PERL script (Schmieder and Edwards, 2011), by truncating bases at the 3′ end with Phred quality score <30;
(3) paired-end read assembly using FLASH (Magoč and Salzberg, 2011), with a minimum overlap of 30 bases and >97% overlap identity.

Taxonomic and diversity analysis were performed with the Metabiote Online v2.0 pipeline (GenoScreen, Lille, France) which is based in part on QIIME software v 1.9.1 (Caporaso et al., 2010). Following pre-processing, full-length 16S rRNA gene sequences were checked for chimera sequences (in-house method based on Usearch 6.1). Similar sequences with a nucleic identity defined threshold (97% identity for an affiliation at the genus level on the V4–V5 regions of the 16S rRNA gene) were clustered with Uclust v1.2.22q (Edgar, 2010) through an open-reference Operational Taxonomic Units (OTU) picking process and complete-linkage method, finally generating groups of sequences or “Operational Taxonomic Units” (OTUs). An OTU cleaning step involving elimination of singletons was performed. The most abundant sequence of each OTU was considered as the reference sequence of its OTU and taxonomically compared to a reference database included in the Greengenes database (release 13_8^1^; DeSantis et al., 2006) by the RDP classifier method v2.2 (Cole et al., 2014). Alpha-diversity metrics (Chao1 index) within samples were computed using QIIME v 1.9.1.

### Bacterial Community Composition Analysis

Univariate statistical analyses (Student test, ANOVA) were performed with XLSTAT (Version 2016.02.27390). Multivariate analyses [principal component analysis (PCA)] were performed within R^2^. Bacterial community composition data obtained from Illumina MiSeq sequencing were analyzed by PCA ordination. Data were first normalized using Hellinger transformation (Legendre and Gallagher, 2001; Ramette, 2007). Explanatory variables consisted of quantitative variables including: CEs concentrations (and sum of total CE) (mg ⋅ L^-1^), pH, redox potential (mV) temperature (°C), sulfate, chloride and acetate concentrations (mg ⋅ L^-1^), gene copies number (copies ⋅ mL^-1^) *pceA*(Dhc), *pceA*(Dhb), *vcrA* and *bvcA* to 16S rRNA gene ratios (%). Explanatory variables were standardized to provide dimensionless variables and remove undue influence of magnitude differences between scales or units. The relationship between community profiles and biogeochemical variables was investigated by fitting environmental vectors *a posteriori* onto the PCA. Their significance was assessed with a 1000-step Monte-Carlo permutation test. Significance was inferred at *p* < 0.05.

### OTU935 Sequencing

16S rRNA gene was amplified with *Dehalococcoides* specific primers using DNA extracted from the 10 μM_PCE_T8 experiment: forward primer Fp DHC 1 (5′-GATGAACGCTAGCGGCG-3′) and reverse primer Rp DHC 1377 (5′-GGTTGGCACATCGACTTCAA-3′) (Hendrickson et al., 2002), by 30 s hybridisation at 66°C, and 3 min elongation at 72°C for 40 cycles. Then, ligation and transformation were realized according to pGEM-T^®^ easy vector (Promega, United States) protocol. After 37°C overnight incubation on LB media, one plasmid was purified according to NucleoSpin^®^ Plasmid Columns protocol (Macherey-Nagel, Germany). Forward and reverse sequencing was realized by Sanger sequencing on ABI3730XL at GenoScreen (France). Consensus sequence was created from forward and reverse sequences and aligned by ClustalW method thanks to BioEdit v7.0.5.3 (Hall, 1999) with 18 NCBI sequences: 17 sequences of the 16S rRNA gene of *Dehalococcoides* strains and *Desulfitobacterium hafniense* DCB-2 strain. Finally, phylogeny was performed on 923 nucleotides with SeaView v4.7 (Gouy et al., 2010) with BioNJ - JC distance method, a 1,000 bootstrap value and without ignoring GAPs.

## Results

### Organohalide Respiration of CEs

Redox potential (*E*_h_) values decreased from -130 mV to -350 mV in all microcosms (data not shown), thus confirming suitable conditions for reductive dechlorination (Löﬄer et al., 2013). The pH in all microcosms remained between 6.9 and 7.9 during the experiment, close to the measured *in situ* Pz6-(10) groundwater pH of 7.0.

**FIGURE 2:**
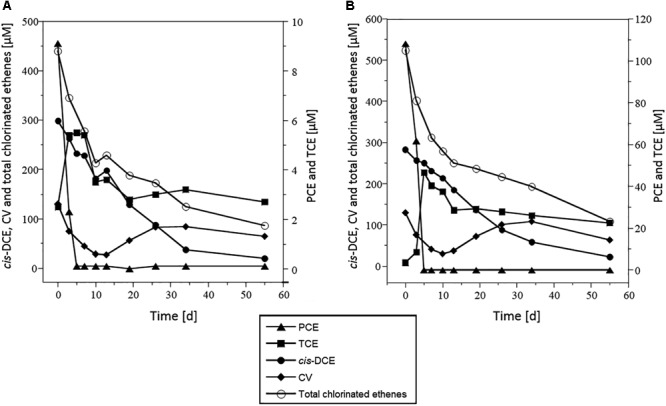
Dissipation of CEs in microcosms spiked with PCE and acetate. Concentrations of PCE (triangles), TCE (squares), *cis*-DCE (circles), VC (diamonds), and total chlorinated ethenes (white circles), in microcosms spiked with 10 μM PCE **(A)** or 100 μM PCE **(B)**. Represented values are the average of three replicates. Error bars are not shown for clarity and were below 100% (see Supplementary Figure S1). Data from microcosms that received acetate but no Na_2_S are shown as a representative example. Results were very similar for other experimental conditions tested.

In all microcosms except killed controls, PCE was nearly completely dissipated within 5 days under both 10 and 100 μM PCE spiking conditions (Figure 2 and Supplementary Figure S2). TCE was only partially dissipated, and no further dissipation was observed after 20 days. In contrast, *cis-*DCE decreased continuously throughout the incubation, down to the detection limit after 60 days for both PCE spiking doses. Transient build-up of VC was observed, with VC first decreasing until day 10, and then increasing until day 35, before decreasing again. This may reflect VC formation from *cis*-DCE dechlorination with only limited VC dissipation until day 35. Sulfate initially present in these microcosms was completely reduced to sulfide after 10 days (Supplementary Figure S3).

**FIGURE 3:**
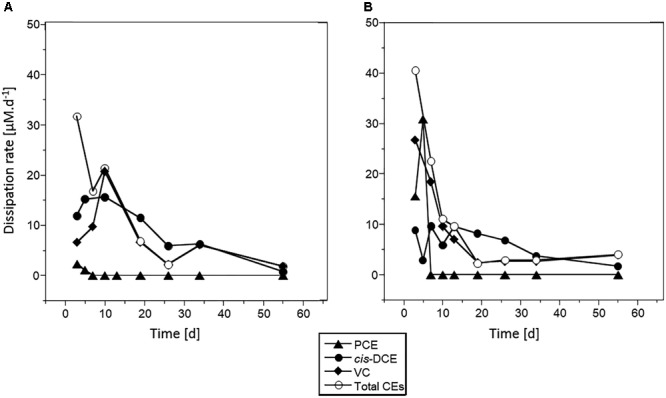
Chlorinated ethene dissipation in microcosms spiked with PCE and acetate. Dissipation rates estimated for PCE (triangles), *cis*-DCE (circles), VC (diamonds), and total chlorinated ethenes (white circles) are given for microcosms spiked with 10 μM PCE **(A)** or 100 μM PCE **(B)**.

Maximum dissipation rates were estimated at (mean ± standard deviation) 30 ± 2 μM ⋅ day^-1^, 10 ± 5 μM ⋅ d^-1^ and 26 ± 5 μM ⋅ d^-1^ for PCE, *cis*-DCE and CV, respectively (Figure 3, results with acetate and no Na_2_S addition). Highest rates were observed over the 10 first days of incubation. The PCE dissipation rate was 10-fold higher in microcosms spiked with 100 μM PCE than in those spiked with 10 μM PCE. For other CEs, similar rates were observed in microcosms with 10 or 100 μM PCE. Dissipation rate of *cis*-DCE was lower than that of PCE (10 ± 5 μM^.^d^-1^) and decreased by 10 μM^.^d^-1^ after 20 days. VC was rapidly dissipated within the first 10 days, with a rate up to 25 μM^.^d^-1^, and then decreased to 5 μM^.^d^-1^ for the remaining incubation time.

Killed controls showed low dissipation of CEs (<20%, data not shown), confirming that dissipation was the result of microorganisms activity in non-sterile microcosms.

Addition of acetate and Na_2_S did not impact PCE dissipation kinetics. All complementary analyses were thus carried out from microcosms amended with acetate only (without Na_2_S, see Figure 2, 3), and all the results presented hereafter were obtained under those conditions.

### Dechlorination Activity and Dehalogenase Gene Abundance

**FIGURE 4:**
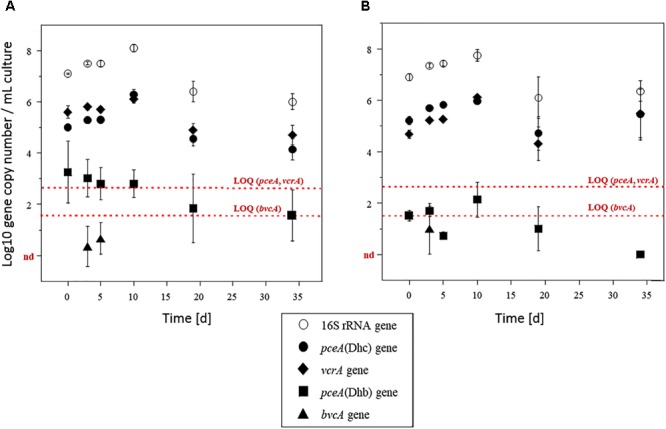
Biomarker genes in microcosms. Quantification of 16S rRNA gene (white circles), *pceA*(Dhc) (diamonds), *pceA*(Dhb) (squares), *vcrA* (black circles) and *bvcA* (triangles) genes in microcosms amended with acetate and spiked with 10 μM **(A)** or 100 μM **(B)** PCE. The *tceA* gene was not detected. Values represent the mean (±standard deviation) of three replicates. Quantification limits of qPCR analysis for functional genes are indicated by red lines. nd, not detected.

Abundance of selected reductive dehalogenase genes (*pceA, tceA, vcrA*, and *bvcA*) and total bacterial abundance (assessed from the 16S rRNA gene) were determined by qPCR (Figure 4). Total bacterial abundance increased from 10^7^ to 10^8^ copies ⋅ mL^-1^ during the first 2 weeks of incubation under both PCE spiking doses, and then remained constant until the end of the experiment. The *vcrA* gene and the version of the *pceA* gene carried by *Dehalococcoides* [*pceA*(Dhc)], initially present at levels around 10^5^ copies ⋅ mL^-1^ also increased by one order of magnitude under both PCE spiking conditions. Notably, increase in abundance of *pceA*(Dhc) occurred during PCE dissipation. Then, after 19 days of incubation, *pceA*(Dhc) gene copies decreased by one order of magnitude before stabilizing around 2.10^4^ and 4.10^4^ copies ⋅ mL^-1^ in microcosms spiked with 10 and 100 μM PCE, respectively. The ratio *pceA*(Dhc)/16S *rrnA* was relatively low at the beginning of the experiment (around 0.1% relative abundance). However, the ratio *pceA*(Dhc)/16S *rrnA* ratio increased during the incubation by at least one order of magnitude, up to 1 and 3.5% for microcosms spiked with 10 μM PCE and 100 μM PCE, respectively (Supplementary Figure S4). Abundance of the *vcrA* gene, potentially involved in the two organohalide respiration steps from *cis*-DCE to ethene, followed the same pattern as that of *pceA*(Dhc) (with a magnitude of about 0.4 log) over the duration of the experiment. The *vcrA*/16S *rrnA* ratio increased from about 2.5% relative abundance at the start of the experiment to 6% in microcosms spiked with 10 μM PCE and 12% in microcosms spiked with 100 μM PCE after 34 days of incubation (Supplementary Figure S4). Overall, changes of the abundance of *pceA*(Dhc) and *vcrA* genes compared to the total community as monitored using 16S rRNA gene as proxy (Figure 4) paralleled the dynamics observed for complete dissipation of *cis*-DCE and VC (Figure 2).

Surprisingly, the dehalogenase gene *pceA*(Dhb) involved in the organohalide respiration of PCE to *cis*-DCE by strains of *Dehalobacter* and *Desulfitobacterium* genera (Regeard et al., 2004) was less abundant than dehalogenases genes *pceA*(Dhc) and *vcrA* by two orders of magnitude (Figure 4). At about 10^3^ copies ⋅ mL^-1^ at the beginning of the experiment, it remained above the limit of quantification (LOQ, 10^2^ copies ⋅ mL^-1^) during the 10 first days of the experiment. It then decreased and remained below the LOQ. Similarly, the absence of detection of *tceA* gene was in keeping with the observed incomplete dissipation of TCE. As for gene *bvcA* involved in VC organohalide respiration, it was only detected at very low copy-numbers, and only during the first 10 days of incubation.

### Bacterial Community During Organohalide Respiration of CEs

Changes in bacterial community were first assessed by CE-SSCP fingerprinting analyses for all microcosm experiments (i.e., with/without acetate, sodium sulfide and/or PCE). Only minor variations were apparent during the first 19 days. Distinct changes were then observed after 34 days (Supplementary Figure S5), depending on the PCE spiking dose.

Principal component analysis of CE-SSCP fingerprints confirmed the observed variations (Supplementary Figure S6). The first two principal components explained 38% of the variability, the first component (24%) being mainly associated with SSCP peaks only present during the first stages of the incubation. *A posteriori* fitting of the main physico-chemical variables and gene abundance data onto the PCA ordination plot suggested that these changes were associated to the decrease of PCE, *cis*-DCE and VC concentrations, and with the increase of *pceA*(Dhc) and *vcrA* dehalogenase gene abundances over time (Supplementary Figure S6). Microcosm samples at the beginning of the incubation (0 and 5 days), and at intermediate (10 days) and late (34 days) sampling times were selected for high-throughput sequence analysis of 16S rRNA gene amplicons to identify bacterial taxa present over time and potentially involved in successive steps of organohalide respiration of CEs.

A total of 412,231 high-quality sequences of the V4–V5 region of the 16S rRNA gene (∼350 bp) were obtained from separately pooled DNA extracted from triplicate microcosms spiked with 10 and 100 μM PCE at the selected four incubation times (0, 5, 10, and 34 days) (Supplementary Table S2). Rarefaction curves did not systematically reach saturation in the 1st days of incubation, although patterns of alpha diversity and overall relative abundances of dominant lineages could be retrieved (Supplementary Figure S8). In the later phase of incubation (10 and 34 days), in contrast, rarefaction curves of diversity indices reached saturation (Supplementary Figure S7), indicating a reduction of bacterial α-diversity through time in laboratory microcosms.

**FIGURE 5:**
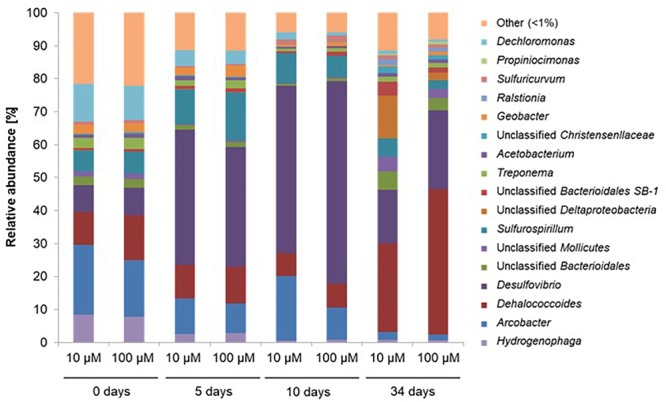
Relative abundance of the 17 most abundant genus-level OTUs during CEs dissipation. Only data obtained for microcosms with acetate amendment were analyzed in detail. A total of 412,231 sequences of the V4–V5 region of the 16S rRNA gene were obtained by Illumina MiSeq and analyzed with Qiime software. 10 μM, 100 μM: concentrations of PCE added. OTUs with <1% abundance were clustered together.

At the start of the experiment, genera *Hydrogenophaga, Arcobacter, Dehalococcoides, Desulfovibrio, Sulfurospirillum*, and *Dechloromonas* dominated the microcosms, and were found in similar proportions at both investigated concentrations of spiked PCE (Figure 5). On average, these genera represented 63 and 65% of the total number of sequences for microcosms spiked with 10 μM PCE and 100 μM PCE, respectively. Dominant genus-level taxa belong to *Proteobacteria*, except *Dehalococcoides* affiliated to *Chloroflexi*. Over the course of the experiment, *Arcobacter* (19.2–2.0%), *Hydrogenophaga* (8.1–0.76%), *Dechloromonas* (10.8–0.8%), and *Geobacter* (2.0–0.4%) strongly decreased under both PCE amendment conditions (Figure 5). On the other hand, several genus-level taxa showed significant increases in relative abundance, e.g., *Desulfovibrio* (8.2–56.0%) and *Dehalococcoides* (11.8–35.6%) (Figure 5).

### Dehalogenation-Associated Taxa

**FIGURE 6:**
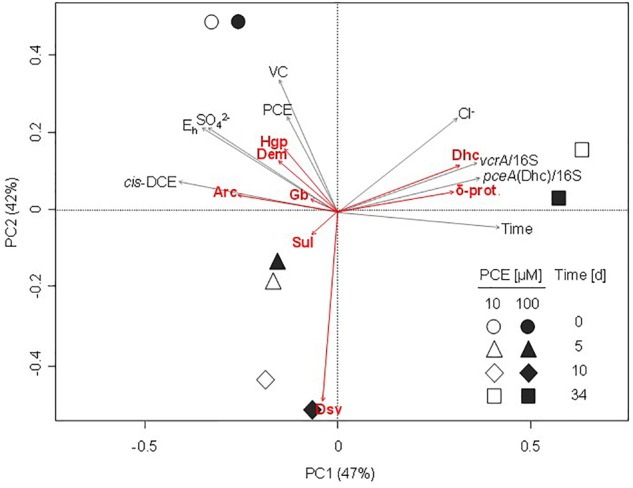
Principal component analysis (PCA) ordination plot of bacterial communities during CEs dissipation. Largest contributions of bacterial taxa are represented by red arrows. Significant physico-chemical and biomolecular explanatory variables were fitted *a posteriori* on the PCA and are represented by gray arrows. Values on the axes indicate the percentage of the total variation explained by the corresponding axis (PC1, principal component axis 1; PC2, principal component axis 2). δ-prot., *Deltaproteobacteria*; Arc, *Arcobacter*; Dem, *Dechloromonas*; Dhc, *Dehalococcoides*; Dsv, *Desulfovibrio*; Gb, *Geobacter*; Hgp, *Hydrogenophaga*; Sul, *Sulfurospirillum*.

High throughput sequencing of the 16S rRNA gene V4–V5 region revealed changes in taxa associated with dehalogenation coinciding with changes in bacterial community diversity (Figure 6). Using PCA analysis, the main PC1 axis explained 42% of community diversity change, and was mainly linked to *cis*-DCE concentration, abundance of *pceA* and *vcrA* genes and occurrence of *Dehalococcoides*. The second PC2 axis explained 30% of the variability in bacterial community diversity, and mainly reflect PCE and VC concentration changes. Only slight differences between microbial community patterns were observed in early stages of the experiment (0–5 days) between microcosms spiked with 10 μM or with 100 μM PCE. As already seen by CE-SSCP fingerprinting analysis, larger differences in community composition were observed at later stages (10–35 days) with both 10 and 100 μM PCE amendments.

Worthy of note, 3 of the 17 most abundant genera at the beginning of the experiment significantly contributing to PCA ordination were potentially associated to reductive dechlorination (Figure 6), e.g., *Dehalococcoides* (10% average abundance), *Sulfurospirillum* (6.4% average abundance), and *Geobacter* (2.7% average abundance). Interestingly, *Sulfurospirillum*-related taxa increased in the early stages of incubation in microcosms spiked with 10 μM PCE (6.3–10.5% average abundance) as well as in microcosms spiked with 100 μM PCE (6.6–14.5% average abundance), and then decreased to about 4% under both PCE conditions. Relative abundance of *Geobacter* remained stable (2.0% average abundance with 10 μM PCE) or slightly increased (2.8–3.5% with 100 μM PCE) and then decreased, especially in microcosms with 100 μM PCE. Relative abundance of *Acetobacterium* taxa, also associated with reductive dehalogenation (Terzenbach and Blaut, 1994), remained stable (1.0% average abundance) at all sampling times and for both PCE amendments. In contrast, other taxa putatively associated to reductive dechlorination (all affiliated to the class of *Dehalococcoidetes*) were only found in minor proportions (<0.3% abundance) throughout the experiment (data not shown). Sequences associated with prominent dehalogenating taxa *Dehalobacter* and *Desulfitobacterium* were not found.

**FIGURE 7:**
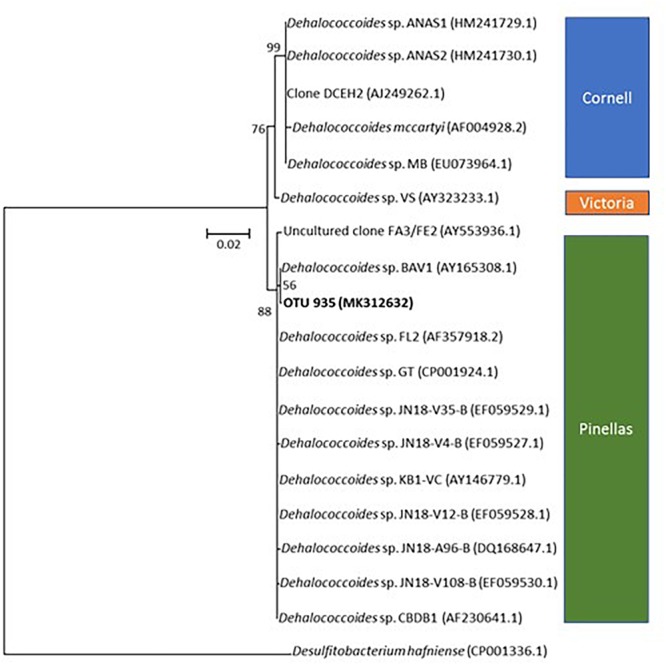
Phylogenetic affiliation of OTU935 within genus *Dehalococcoides*. The tree was obtained by the Neighbor-Joining and Jukes and Cantor methods from a 923 nucleotides alignment of 16S rRNA gene sequences. OTU935 of the microcosm is displayed in bold. *Dehalococcoides* subgroup affiliation is also shown. Bootstrap values are expressed as the percentage of 1000 replications.

One of the most abundant taxa identified in microcosms is affiliated to *Dehalococcoides*, and more specifically to the *Dehalococcoides* Pinellas group (Figure 7) (Hug and Edwards, 2013). As Miseq sequencing do not allow accurate taxa affiliation down to genus level, the affiliation to Pinellas was confirmed by 16S rRNA gene sequence phylogeny on 923 nucleotides of the corresponding OTU (Genbank MK312632). Relative abundance of this taxon remained stable (about 10% abundance) in the initial phase of the experiment (see Figure 5), independently of PCE amendment. A subsequent decrease to 7% average abundance across microcosms was noted under both conditions, followed by a significant (four–sixfold) increase of *Dehalococcoides* abundance at a later stage (Figure 5). This paralleled the observed increase in *pceA*(Dhc) and *vcrA* genes found in *Dehalococcoides* (Figure 6).

## Discussion

The main incentive for this study was to investigate whether laboratory microcosm studies can help identify specific dehalogenation reactions and associated bacterial taxa in groundwater contaminated with CEs that could then be stimulated for bioremediation. Abundances and dynamics of key dehalogenase genes (*pceA, tceA, vcrA*, and *bvcA*), bacterial community composition and dehalogenation-specific taxa through the 16S rRNA gene as proxy were thus inventoried in laboratory microcosms of groundwater contaminated with multiple chlorinated solvents (Table 2).

The experiment specifically focused on effects of PCE exposure (10 and 100 μM) on dissipation rates and abundances of selected biomarkers. Complete dissipation of PCE and *cis*-DCE (in sampled site water and following *in labo* PCE dissipation), as well as partial dissipation of TCE and VC, were observed in all microcosm experiments. Only minor dissipation of CEs was observed in killed controls, confirming the prevailing role of microorganisms. Notably, organohalide respiration occurred rapidly and independently of acetate and/or addition of a reducing agent (Na_2_S). This confirms that reductive dehalogenation likely proceeded through hydrogen supplied through the 5% H_2_/N_2_ atmosphere as the electron donor, and that sufficient carbon was available for biomass production from the groundwater and the preculture used as inoculum.

Monitoring of key reductive dehalogenase genes in microcosms showed a correlation with CE dissipation. It also suggested the dominant involvement of the “Pinellas” subgroup of the *Dehalococcoides* genus (Hug and Edwards, 2013) in the process (Figure 7). Dehalogenase genes *pceA*(Dhc) and *vcrA* were already abundant at the start of the experiment (Figure 4). Groundwater bacteria carrying these genes were likely enriched in the activated preculture prepared from Pz6(10) groundwater and used as inoculum. Upon laboratory incubation, *pceA*(Dhc) and *vcrA* dehalogenase genes abundance increased by one order of magnitude in the initial phase of the experiment, with concomitant complete dissipation of PCE and partial dissipation of *cis*-DCE and VC. This is in agreement with previous studies (Amos et al., 2008; Ise et al., 2011; Aktaş et al., 2012; Baelum et al., 2013). In addition, increase in the 16S rRNA gene suggested selective growth of dehalogenating strains during dechlorination of CEs (Figure 4). In the later phase of the experiment, no further increase of genes *pceA*(Dhc) and *vcrA* was observed (Figure 4), while *cis*-DCE and VC dissipation continued (Figure 2). This suggested that in the presence of hydrogen, dechlorination was supported by sufficient abundance of bacteria containing *vcrA*, as reported previously (Cupples et al., 2004).

*Dehalococcoides* strains may carry several different dehalogenase genes (Behrens et al., 2008). Furthermore, their co-occurrence in CE-degrading enrichment cultures derived from organohalide-contaminated sites has already been observed (Scheutz et al., 2008; Baelum et al., 2013; Kranzioch et al., 2015). Very similar variations and gene copy numbers observed for *pceA*(Dhc) and *vcrA* (Figure 4) suggest that these genes are associated with the same bacteria. However, *pceA*(Dhc) and *vcrA* genes are usually found in different strains. *Dehalococcoides* sp. strain VS, the only bacterium reported to date that carries both *pceA* and *vcrA* genes (Muller et al., 2004; Behrens et al., 2008; Lee et al., 2008), is able to dechlorinate PCE to TCE or *cis*-DCE to ethene, but not TCE to *cis*-DCE (Lee et al., 2008). A comparable situation was encountered here, with complete dehalogenation of PCE and *cis*-DCE but only partial dissipation of TCE (Figure 2). However, the only *Dehalococcoides*-related OTU identified in microcosms by high-throughput sequencing (Figure 5) is affiliated to the Pinellas group and therefore appears phylogenetically distinct from that of the VS strain, which belongs to the Victoria group (Nishimura et al., 2008) (Figure 7). These results, together with patterns of dehalogenase genes *pceA* and *vcrA*, lead to the hypothesis that a new strain of a dehalogenating *Dehalococcoides* sp. containing both *pceA*(Dhc) and *vcrA* genes is involved in organohalide respiration of CEs in groundwater from the Themeroil site.

Abundance data of other dehalogenase genes also allows some conclusions to be drawn. For instance, low abundance of genes *pceA*(Dhb) and *bvcA* suggests an only minor involvement of PCE- and TCE-degrading bacteria carrying these dehalogenases, such as *Desulfitobacterium* and *Dehalobacter* strains (Suyama et al., 2002; Daprato et al., 2007; Rupakula et al., 2013). Similarly, *Dehalococcoides* phylotypes identified in this study likely did not carry the *bvcA* gene for VC organohalide respiration, unlike other known representatives of this genus (Krajmalnik-Brown et al., 2004). Finally, failure to detect the *tceA* gene, together with TCE accumulation in microcosms, suggests the absence of TCE-dehalogenating *Dehalococcoides* sp. Worthy of note, this contrasts with the low TCE concentration (Table 1), and detection of genes *pceA*(Dhb) and *tceA* in groundwater when measured directly on-site (data not shown). Hence, growth of *tceA*-containing bacteria may be limited under the chosen laboratory microcosm conditions.

Investigations of the dynamics of bacterial composition as a function of CE organohalide respiration in microcosms may help to associate observed patterns of dehalogenation with bacterial taxa identified in microcosms. CE-SSCP fingerprinting analyses proved useful for initial characterisation of bacterial community dynamics in microcosm experiments, and allowed to select key samples for taxonomic characterisation of communities by Illumina MiSeq sequencing of 16S rRNA gene amplicons. Despite being less precise than sequencing, CE-SSCP analysis yielded a similar picture of the main determinants shaping the evolution of the bacterial community in microcosms (Supplementary Figure S6) to high-throughput sequencing (Figure 5).

Overall, microcosms sustained diverse bacterial populations capable of different terminal electron-accepting processes. Bacterial communities were dominated by *Proteobacteria* and *Chloroflexi*, as previously reported for chlorinated hydrocarbon-contaminated groundwater (Abbai and Pillay, 2013; Kotik et al., 2013). A large proportion of recovered microorganisms was putatively associated with iron and sulfate reduction, in agreement with physico-chemical conditions in microcosms (Table 1). Only a few dominant taxa significantly contributed to the overall change of bacterial communities over time.

Regarding taxa associated with dehalogenation, increasing proportions of *Dehalococcoides* were observed (Figure 5). In contrast, the proportion of *Sulfurospirillum* and *Geobacter*, known to inhabit contaminated aquifers (Duhamel and Edwards, 2006; Maillard et al., 2011; Kranzioch et al., 2013; Lee et al., 2015), decreased throughout the experiment. These two genera include members capable of respiratory reductive dehalogenation of PCE, and may thus have contributed to dechlorination of PCE to TCE and DCE at the very beginning of the experiment. Furthermore, *Sulfurospirillum* and *Geobacter* strains capable of reductive dehalogenation of PCE to *cis*-DCE usually contain *pceA* genes more closely related to *pceA*(Dhb) of *Dehalobacter* and *Desulfitobacterium* strains (Neumann et al., 1998; Wagner et al., 2012; Buttet et al., 2013), which was detected in microcosms in early stage of the experiment. But more likely, as these taxa are non-obligatory organohalide-respirers (Wagner et al., 2012; Goris et al., 2014), they could also have used alternative endogenous electron acceptors, such as Fe(III) initially present in the site groundwater, and produced hydrogen necessary to *Dehalococcoides* to go further in the organohalide respiration pathway. This is likely to have occurred since recovered sequences associated with these OTUs were affiliated to non-dehalogenating strains of these genera (Sung et al., 2006a; Goris et al., 2014). Members of these taxa can also produce corinoïd cofactors that could be used for *Dehalococcoides* growth (Yan et al., 2012).

In addition, OTUs related to *Acetobacterium*, a genus including strains degrading PCE to TCE (Terzenbach and Blaut, 1994), were systematically found in low abundance (<1%) throughout the experiment. Although data obtained so far do not allow to conclude on their involvement in PCE dehalogenation in the present study, strains of *Acetobacterium woodii* have been reported to produce vitamin B12, an essential co-factor for reductive dechlorination (He et al., 2007). Hence, their presence could potentially benefit dissipation of CEs by reductive dehalogenation. Identifying taxa in groundwater microcosms whose activity would depend on the presence of CEs would require additional experiments as well as complementary approaches, such as, e.g., stable isotope probing (Chen and Murrell, 2010).

Operational Taxonomic Units corresponding to other genera often associated with reductive dehalogenation, such as *Desulfitobacterium, Dehalobacter* (Maillard et al., 2011; Rouzeau-Szynalski et al., 2011) and *Dehalogenimonas* (Maness et al., 2012) were detected at very low abundances, as expected from the low abundance of dehalogenase *pceA*(Dhb) typical of these genera (Regeard et al., 2004). In addition, these genera are not necessarily associated to organohalide respiration of CEs (Holscher et al., 2004; Dugat-Bony et al., 2012). Increase in abundance of an unclassified taxon associated with *Deltaproteobacteria* also occurred in the later phase of incubation (Figure 5). Indeed, *Deltaproteobacteria* are often associated with key dechlorinators in contaminated groundwater (Kotik et al., 2013; Adetutu et al., 2015; Kaown et al., 2016).

Some taxa, not usually associated with dehalogenation, also showed large changes in abundance in our experiments. *Hydrogenophaga, Treponema*, and *Arcobacter*, observed previously in contaminated groundwater (Kotik et al., 2013; Lee et al., 2015), decreased after the early stages of incubation (5 days). This could result from detrimental effects of microcosm conditions on their growth, such as the toxicity of sulfide produced by sulfate-reducing bacteria in the initial phase of microcosm experiments (Supplementary Figure S2). Indeed, the main changes in bacterial communities other than those associated with dehalogenation occurred for taxa related to sulfate reduction, in particular *Desulfovibrio* spp. (Barton and Fauque, 2009). The increase in relative abundance of sulfate reducers, such as strains of the genera *Desulfovibrio, Desulfobulbus*, and *Desulfomicrobium* (Kleikemper et al., 2002), suggests that they grew during the early stages of the experiment. Reductive dehalogenation of CEs is known to compete with sulfate reduction for electron donors and in particular for hydrogen (Davis et al., 2002; Aulenta et al., 2007, 2008). It is thus interesting that both reductive dehalogenation and sulfate reduction occurred in the early phases of the microcosm experiments, suggesting that its establishment was possible due to modest competition between these two metabolisms.

The only OTU associated with *Dehalococcoides* increased in abundance in the later stages of the experiment, which coincided with higher relative abundances of *pceA* and *vcrA* (Figure 4). An almost twofold increase in abundance of this *Dehalococcoides* OTU in microcosms spiked with 100 μM PCE compared to those amended with 10 μM further suggested that it was involved in PCE utilization for growth. Increase in *Dehalococcoides* following a decline of sulfate-reducing bacteria (mainly *Desulfovibrio*) and the decrease of sulfate in microcosms (Figure 5 and Supplementary Figure S3) suggests that relative abundance of sulfate-reducing bacteria, reduction of sulfate and development of *Dehalococcoides* may be related. Possibly, hydrogen transfer between *Desulfovibrio* and *Dehalococcoides* strains upon sulfate depletion may be occurring under these conditions (Drzyzga et al., 2001; Men et al., 2012).

Principal component analysis allowed integrative analysis of chemical and biomolecular data (Figure 6), and how functional gene abundance, bacterial diversity and CEs dissipation may be correlated. Microcosms were mainly discriminated according to the concentration of pollutants PCE, *cis*-DCE and VC (*p* < 0.05) (Figure 6), and time (Supplementary Figure S6). Overall, no clear differentiation of the bacterial community for most of the identified taxa was observed, although a correlation with time, pollutant concentrations or physico-chemical parameters such as redox potential and sulfate concentrations could be identified for a few of them (Figure 6). For example, relative abundance of *Arcobacter, Dechloromonas*, and *Hydrogenophaga* associated OTUs was correlated with the early stages of incubation, together with higher concentrations of CEs, sulfate and redox potential (Figure 5, 6). Several other genera such as *Geobacter, Treponema*, and *Sulfurospirillum* were also linked to the early stages of incubation (Figure 5) and physico-chemical conditions at the beginning of the experiment (Figure 6). In contrast, high relative abundance of taxa related to *Desulfovibrio* correlated to a later incubation time (10 days) (Figure 5).

Regarding dehalogenation, low relative abundance of *pceA*(Dhb) in microcosms was correlated with high concentrations of PCE, thus questioning its association with PCE dissipation. Similarly, abundance of *bvcA* was not significantly associated with changes in physico-chemical parameters or taxonomy. Key shifts observed in bacterial community composition were thus clearly associated with relative abundance of dehalogenase genes *pceA*(Dhc) and *vcrA*, and negatively or not correlated to pollutant concentrations (Figure 6), as expected for genes involved in pollutant transformation. In addition, chloride, sulfate and redox potential were correlated with abundances of *Dehalococcoides*-associated OTUs identified as a potential biomarker of organohalide respiration of CEs in microcosms of groundwater from the contaminated site of interest, as expected for reductive dehalogenation metabolism.

## Conclusion

In summary, an active dechlorinating bacterial community was evidenced and characterized in groundwater samples from the contaminated Themeroil site. Molecular investigations of groundwater microcosms allowed to assess changes of functional genes associated with organohalide respiration of CEs and associated bacterial community composition. Analysis of the relationship between key dehalogenase genes and taxonomic profiling highlighted the importance of specific genera associated with dehalogenation of PCE, *cis*-DCE and VC, as dehalogenation of CEs. Concomitant changes in bacterial community composition revealed different compositions through time, and changes in *Dehalococcoides* and sulfate-reducing bacteria.

Taken together, our results provide new evidence that endogenous *Dehalococcoides* sp. in multi-contaminated groundwater from the investigated site of interest predominantly grows through CE organohalide respiration under anoxic conditions. This, together with patterns of *pceA*(Dhc) and *vcrA* genes, led to hypothesize that a potentially novel *Dehalococcoides* sp. taxon belonging to the Pinellas subgroup and containing both *pceA*(Dhc) and *vcrA* genes is related to dissipation of PCE, *cis*-DCE and VC. This hypothesis remains to be further examined by isolation of dehalogenating strains and experiments in pure cultures. Metagenome sequencing on groundwater samples of the site as well as attempts at isolation and characterisation of this strain in pure cultures and by multi-element compound specific isotope analysis (CSIA) may also help to identify biological pathways and genes associated with dissipation of CEs at the Themeroil site.

## Author Contributions

LH contributed to the experimental design, carried out experimental work, data analysis, and drafted the paper. JH, CJ, and SV contributed to the experimental setup, data analysis, and paper drafting and revision. JD and SF carried out metagenomics sequencing, data analysis, and paper revision. GI contributed to data analysis and paper drafting and revision. CU carried out cloning, sequencing and phylogenetic analyses and paper revision.

## Conflict of Interest Statement

The authors declare that the research was conducted in the absence of any commercial or financial relationships that could be construed as a potential conflict of interest.

## Footnotes

1 http://greengenes.secondgenome.com/

2 http://www.R-project.org
