# Supplementary material for: Functional Genes and Bacterial Communities During Organohalide Respiration of Chloroethenes in Microcosms of Multi-Contaminated Groundwater
Source: Front Microbiol. 2019 Feb 12;10:89. doi: 10.3389/fmicb.2019.00089 (PMC6379275; doi:10.3389/fmicb.2019.00089)
Supplement: Supplementary file 1 [file Table_1.pdf]

Supplementary Material

Functional genes and bacterial communities during organohalide respiration of chloroethenes in microcosms of multi-contaminated groundwater

Louis Hermon, Jennifer Hellal, Jérémie Denonfoux, Stéphane Vuilleumier, Gwenaël Imfeld, Charlotte Urien, Stéphanie Ferreira and Catherine Joulian*

*** Correspondence:** Catherine Joulian: c.joulian@brgm.fr

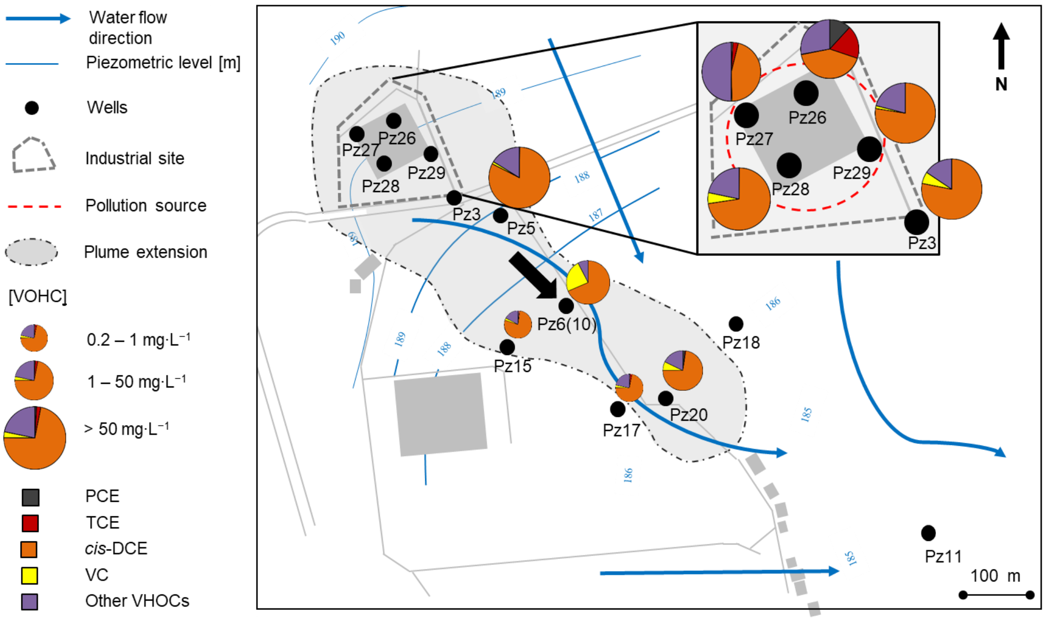

Figure SI-1. Location of the well Pz6(10) at the Themeroil site (Varennes-le-Grand, France). The investigated well is indicated by the arrow. Average concentrations and relative proportions of CEs and others halogenated VOC in June 2015 are shown.

A

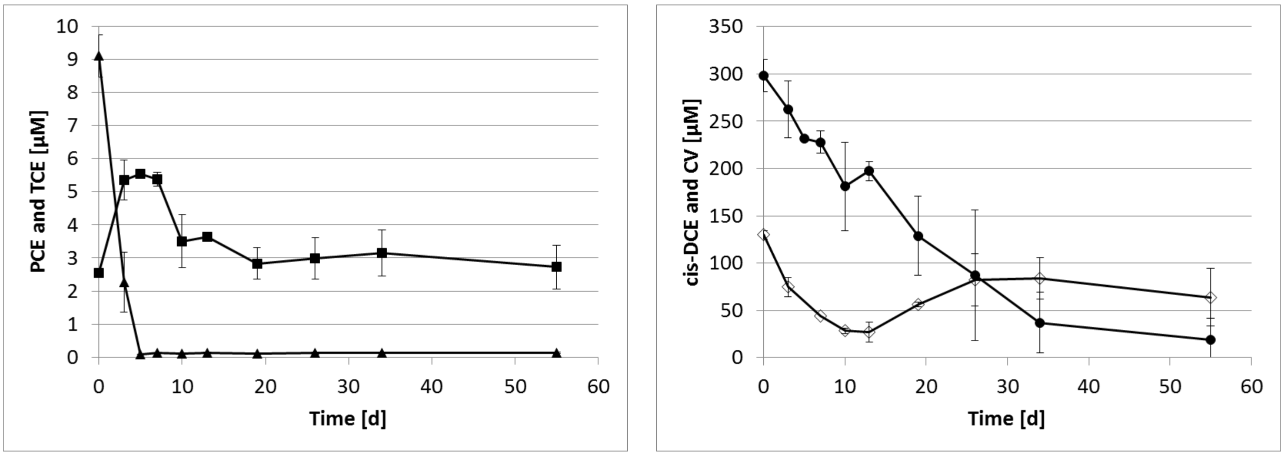

B

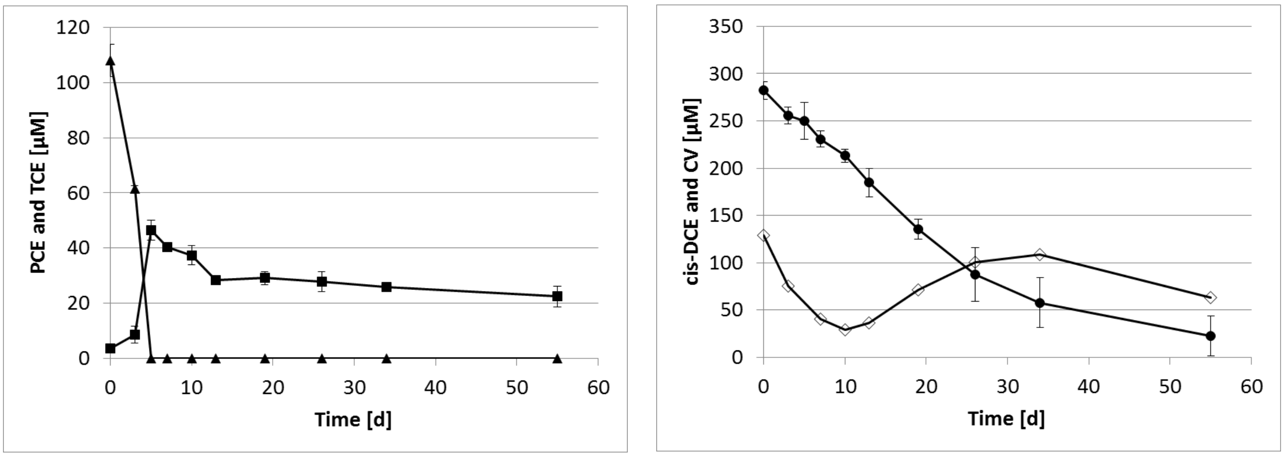

Figure SI-2. Dissipation of CEs in microcosms spiked with PCE and acetate. Concentrations of PCE (triangles), TCE (squares), *cis*-DCE (circles), VC (diamonds), in microcosms spiked with 10 µM PCE (A) or 100 µM PCE (B). Error bars are for three replicates. Data from microcosms that received acetate but no Na_2S are shown as a representative example of the obtained results. Results were very similar under the other experimental conditions tested.

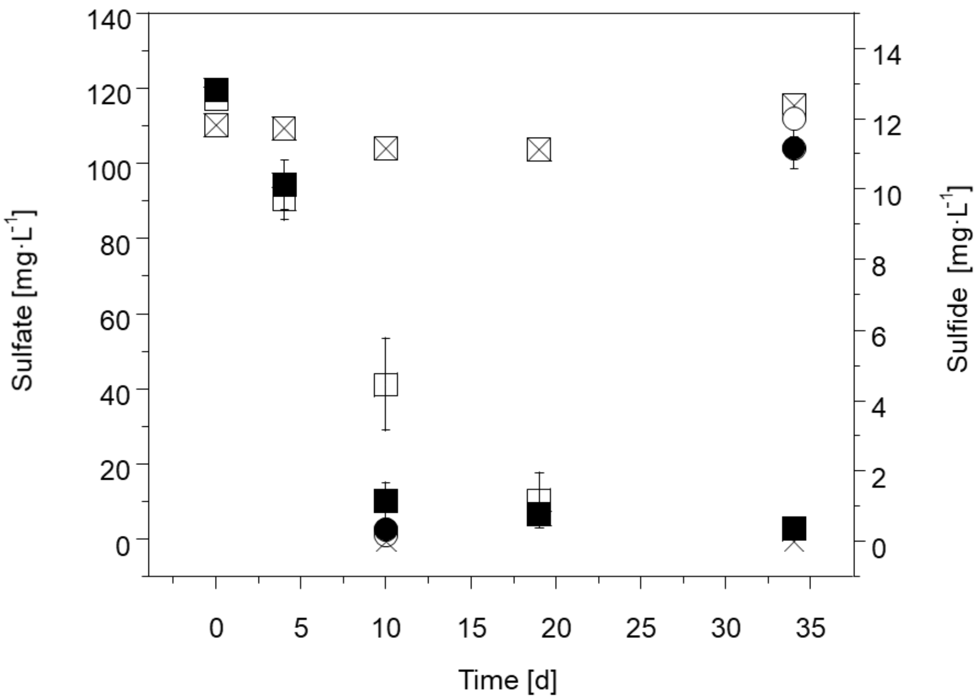

Supplemental Figure SI-3. Sulfate and sulfide concentrations in microcosms. Sulfate (squares) and sulfide (circles) were measured throughout the experiment in microcosms amended with acetate and spiked with 10 µM PCE (open symbols), 100 µM PCE (full symbols) and in killed controls spiked with 10 µm PCE (crosses). Values represent the mean of three replicates (error bars : standard deviation).

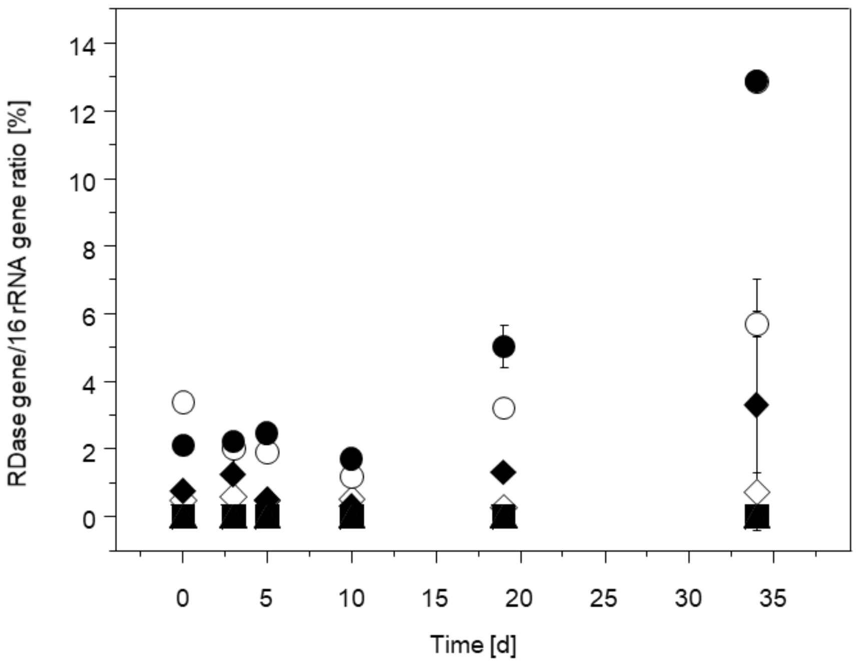

Supplemental Figure SI-4. Relative abundance of dehalogenase genes in microcosms. Time-course of the abundance of *pceA*(Dhc) (diamonds), *vcrA* (circles), *pceA*(Dhb) (squares) and *bvcA* (triangles), relative to that of the 16S rRNA gene, in microcosms spiked with 10 µM PCE (open symbols) and 100 µM PCE (full symbols) and amended with acetate. Values represent the mean of three replicates (error bars : standard deviation).

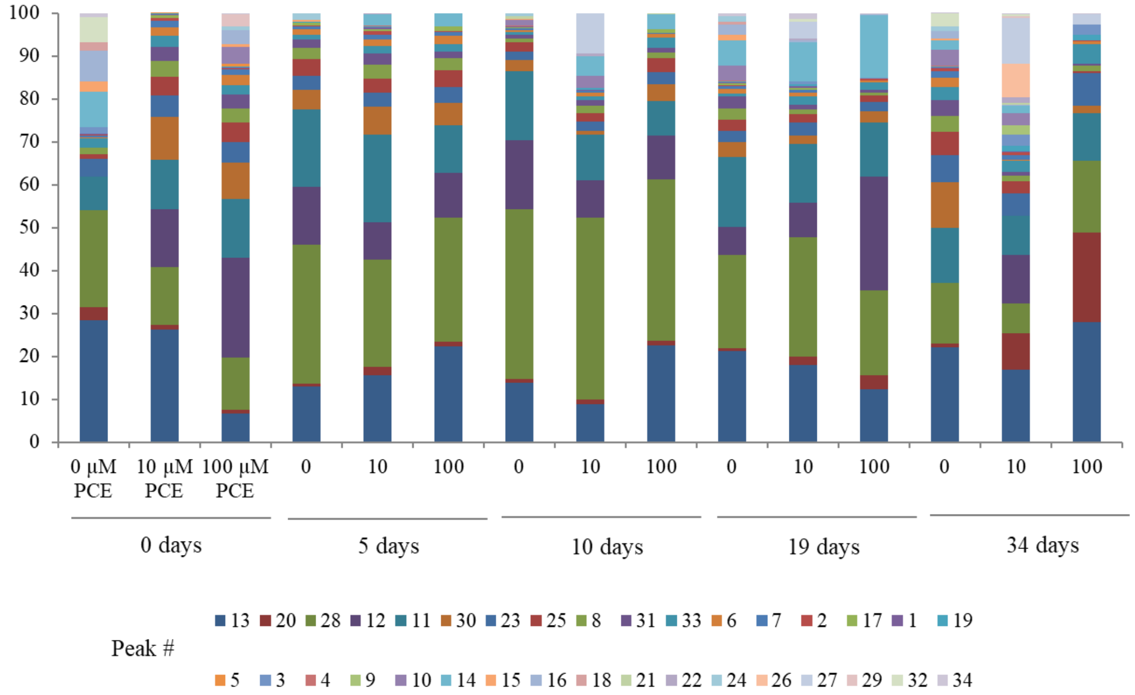

Supplemental Figure SI-5. CE-SSCP of bacterial community in microcosms. Numbers correspond to the total 34 SSCP peaks identified. Relative proportions of peaks, corresponding to ensembles of different bacterial taxa, were calculated from peak areas. Data are presented for acetate-amended microcosms spiked with 10 µM or 100 µM PCE, or microcosms to which no PCE was added, and 5 sampling times (0, 5, 10, 19, 34 days).

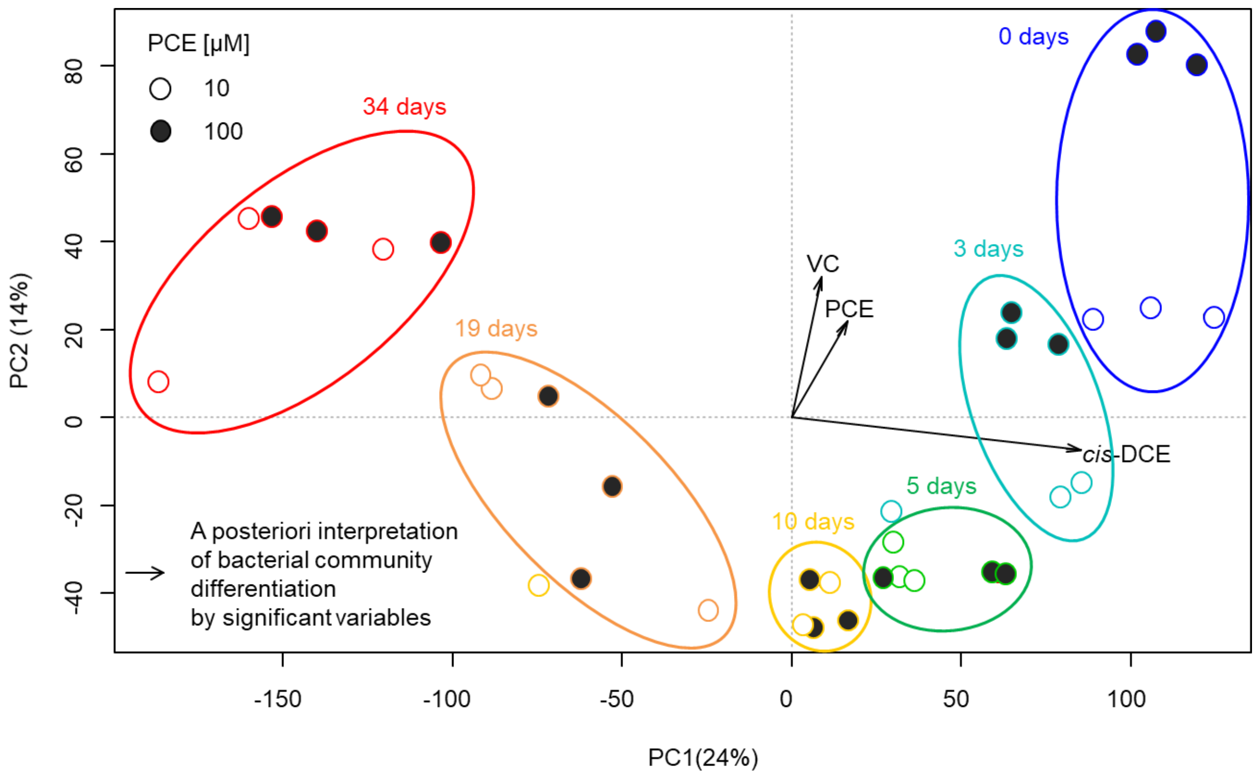

Supplemental Figure SI-6. PCA ordination of bacterial communities in microcosms from CE-SSCP patterns. Data for three microcosms spiked with 10 µM (white circles) and 100 µM (black circles) of PCE over time are presented. Objects colors represent different microcosms sampling times. Vectors represent a posteriori fitting onto the PCA of CE concentration variables explaining bacterial diversity differentiation in microcosms.

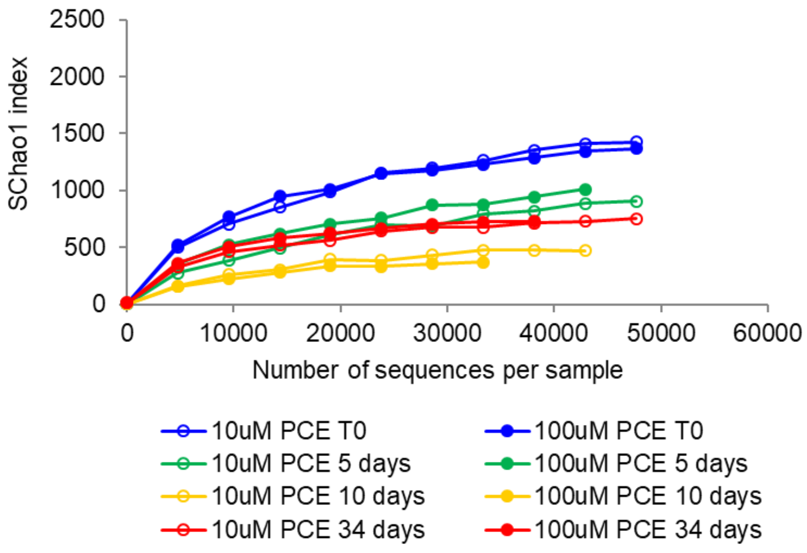

Supplemental Figure SI-7. Bacterial diversity in microcosms. Rarefaction curves for sequences of V4-V5 hypervariable region amplicons of the 16S rRNA gene obtained by Illumina sequencing at timepoints 0 (blue), 5 (cyan blue), 10 (yellow), 34 (red).

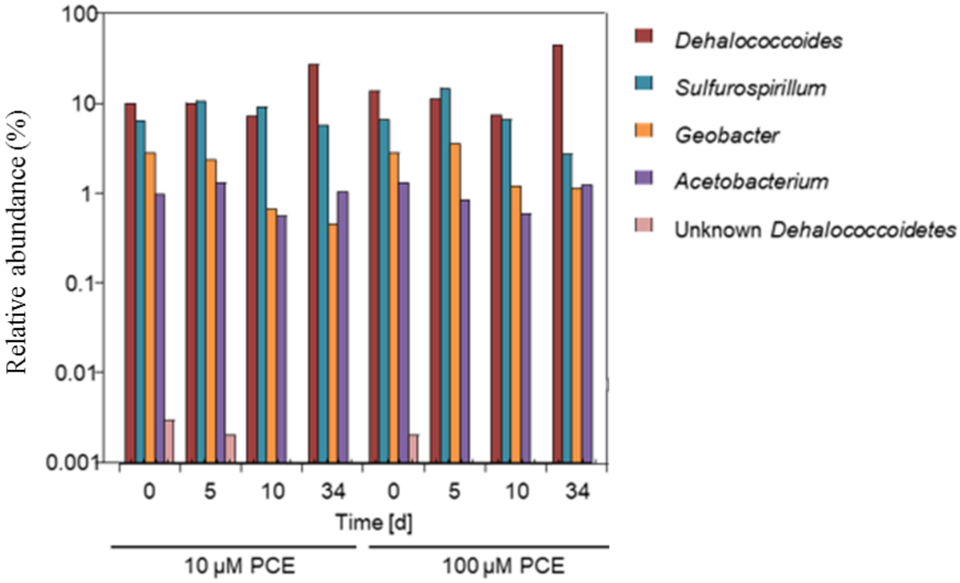

Supplemental Figure SI-8. Relative abundance of dehalogenation-associated taxa.

Supplemental Table SI-1. qPCR standard curves.

Target gene	Equation	R^2	Efficiency [%]	LOQ[a]	Cq NTC[b]
16S rRNA	$-3.63x + 37.65$	0.99	88.6	$4.8 \cdot 10^2$	28.8
pceA(Dhc)	$-3.44x + 37.50$	0.99	94.9	$1.0 \cdot 10^2$	28.0
pceA(Dhb)	$-3.53x + 35.54$	0.98	91.8	$1.0 \cdot 10^2$	No
tceA	$-3.69x + 36.12$	0.99	86.4	$1.0 \cdot 10^1$	No
vcrA	$-3.26x + 34.39$	0.99	102.7	$1.0 \cdot 10^1$	No
bvcA	$-3.45x + 34.67$	0.99	94.8	$1.0 \cdot 10^1$	No

[a] Limit of quantification of the standard curve [copy number/reaction]

[b] Cycle threshold of the no template control (number of amplification cycles)

Supplemental Table SI-2. Number of sequences obtained from Illumina MiSeq amplicon sequencing of 16S rRNA gene V4-V5 region in microcosms.

**Microcosm**	**10 µM PCE**	**100 µM PCE**
0 day	57201	50213
10 days	47146	48860
19 days	57254	54286
34 days	47224	50047
Total (microcosm)	208825	203406
Total (region V4-V5 of 16S rRNA gene)	412231	
